# Contact and Gastric Effect of Peppermint Oil on Selected Pests and Aphid Predator *Harmonia axyridis* Pallas

**DOI:** 10.3390/molecules28124647

**Published:** 2023-06-08

**Authors:** Janina Gospodarek, Agnieszka Krajewska, Iwona B. Paśmionka

**Affiliations:** 1 Department of Microbiology and Biomonitoring, University of Agriculture, al. A. Mickiewicza 21, 31-120 Krakow, Poland; iwona.pasmionka@urk.edu.pl; 2 Department of Biotechnology and Food Science, Lodz University of Technology, 90-530 Lodz, Poland; agnieszka.krajewska@p.lodz.pl

**Keywords:** black bean aphid, Colorado potato beetle, ladybugs, essential oil, non-chemical control, peppermint EO composition

## Abstract

Peppermint essential oil (EO) has been extensively tested to date in reducing stored-product insects and insects of public health concern with very promising results, while only a few studies target important crop pests. There is also very little information on the effects of peppermint EO on non-target organisms, especially concerning contact and gastric effects at the same time. The goal of the investigation was the determination of the effect of peppermint EO on the mortality of *Aphis fabae* Scop.; the feeding intensity and weight gain of *Leptinotarsa decemlineata* Say. larvae; and the mortality and voracity of non-target organism *Harmonia axyridis* Pallas larvae. Our research indicates promising use for the *M. piperita* EO against aphids and young larvae (second instars) of the Colorado potato beetle. *M. piperita* EO showed good insecticidal efficacy against *A. fabae* with LC_50_ = 0.5442% for nymphs and 0.3768% for wingless females after 6 h. Over time, the LC_50_ value decreased. For the second instar larvae of *L. decemlineata*, the LC_50_ values were 0.6278%, 0.3449%, and 0.2020% after 1, 2, and 3 days of the experiment, respectively. On the other hand, older larvae (fourth instar) were characterized by significant resistance to the tested oil concentrations with LC_50_ value = 0.7289% after 96 h. *M. piperita* oil (contact and gastric effects) at a concentration of 0.5% was found to be toxic to young larvae (2 and 5 days old) of *H. axyridis*, while EO at a concentration of 1% was toxic to 8-day-old larvae. Thus, for the sake of ladybug safety, it would be advisable to use EO from *M. piperita* against aphids at concentrations lower than 0.5%.

## 1. Introduction

Essential oils (EOs), used for pest control since ancient civilizations [1], can be an effective alternative to chemical insecticides, especially in areas such as public health insect control [2,3,4,5,6,7], food storage [8,9], veterinary [10,11], and crop protection [12]. They may find wider applications, especially in the context of the emphasis placed on the sustainable use of plant protection products and the promotion of integrated pest management. Reducing the use of chemical pesticides is also one of the main goals of the European Green Deal and Farm to Fork strategy [13,14].

Peppermint *Mentha × piperita* EOs have been extensively tested to date in reducing stored-product insects [15,16,17,18,19,20,21,22] and insects of public health concern, e.g., mosquitos [23,24], household ants [25], houseflies [26,27,28], horse flies [29], lice, and flies that infest water buffaloes [30]. In most of the cases analyzed, the authors indicated the significant effectiveness and usefulness of peppermint oil.

As for crop pests, the effects of peppermint EO on *Bactrocera oleae* Rossi (Diptera: Tephritidae) [31], *Aphis punicae* Passerini (Hemiptera: Aphididae) [32], cotton aphid *Aphis gossypii* Glover (Hemiptera: Aphididae) [33], citrus mealybug, *Planococcus citri* Risso (Hemiptera: Pseudococcidae) [34,35], vine mealybug, *Planococcus ficus* (Signoret) (Hemiptera: Pseudococcidae) [36], Queensland fruit fly *Bactrocera tryoni* Froggatt [37], mushroom cecid flies (Diptera: Cecidomyiidae) [38], and cabbage looper *Trichoplusia ni* Hubner (Lepidoptera: Noctuidae) [39] were analyzed. The effects obtained depended on the dose used, the formulation (crude oil, nanoemulsion), the mode of action (fumigation, gastric, contact), and the pest stage tested. In general, peppermint is indicated for crop protection as a promising source of bioactive substances with insecticidal activity [40]. This plant, used as a component in intercropping, contributed to reducing *Drosophila suzukii* Matsumura adult emergence from fruit compared to conventional ryegrass/clover mixes and also supported greater beneficial insect abundance (predators and pollinators) [41].

The black bean aphid (*Aphis fabae* Scop.) is one of the most dangerous oligophagous pests of crop plants. Its occurrence leads to reduced growth, decreased yield, and even plant death. It is also a vector of many plant viral diseases [42]. Under central and eastern European climate conditions, its main natural enemy is currently the Asian lady beetle (*Harmonia axyridis* Pallas) [43]. The Colorado potato beetle is the main pest of *Solanum tuberosum* plants in most potato-growing regions of the world. Foraging by larvae and adults leads to the complete defoliation of plants. Recent research results indicate the developing resistance of this pest to many classes of insecticides, making the search for alternative means of controlling this pest all the more urgent [44]. 

Despite the importance of the above-mentioned pests, only a few studies using essential oils, particularly peppermint oil, target these insects. Jahan et al. [45], testing the short-term (24 h) fumigative effect of five EOs against *A. fabae*, pointed to peppermint oil as showing the strongest deterrent effect. In contrast, Sajfrtova et al. [46], testing isolate volatile compounds from savory (*Satureja hortensis* L.), thyme (*Thymus vulgaris* L.), lavender (*Lavandula angustifolia* L.), and peppermint (*Mentha piperita* L.), as well as EOs from these plants against the larvae of *L. decemlineata*, indicated the lowest LD_50_ for savory EO. 

The high insecticidal efficacy of EO against pest insects implies the need to evaluate the action of this substance against non-target organisms, many of which are natural enemies of pests or have other important roles in the environment [47]. So far, only a few studies have been devoted to the effects of peppermint EO on beneficial invertebrates. Significant biochemical and physiological effects on honeybee workers have been demonstrated when exposed to peppermint EO either by oral or contact treatments, with significantly higher toxicity found for nanopreparations compared to crude materials [48]. On the other hand, a study on the effect of EOs from different plants on the biological and reproductive parameters of parasitoid *Trichogramma galloi* Zucchi (Hymenoptera: Trichogrammatidae) showed that peppermint was one of three plant species out of the 10 analyzed in total that were selective towards this beneficial insect. Accordingly, the authors indicated that it could be included in integrated pest management programs for this parasitoid [49]. There is no information in the available literature on the effects of peppermint EO on the Asian lady beetle. A contact toxicity test of *M. piperita* EO on the larvae of another ladybug species, *Coccinella undecimpunctata*, showed that the LC_50_ was approximately four-fold greater for EOs towards the predator than towards aphids, suggesting its safety for non-target insects [32]. In general, studies of non-target organisms involve contact effects (i.e., the test insect is sprayed with an oil solution) [50,51] or fumigation (the insect is exposed to the oil in an enclosed space) [52]. The approach proposed in this paper—a contact and gastric effect at the same time (i.e., the food offered to the predator is also treated with EOs)—is more similar to the conditions we may encounter in the field following the application of EOs, where the predator can feed on aphids coated with oil. In addition, the effect of extracts on non-target organisms is not necessarily death; the effect may also manifest itself in changes in voracity [53].

The goals of the investigation were: (a) the determination of the effect of EO extracted from dried peppermint on the mortality, feeding intensity, and weight gain of selected pests, i.e., nymphs and wingless females of the black bean aphid and L2 (second instar) and L4 (fourth instar) larvae of Colorado potato beetle, respectively; (b) an examination of the effect of peppermint EO on the mortality and voracity of non-target organism *H. axyridis* larvae at different ages.

## 2. Results

### 2.1. Chemical Composition of EO

Peppermint EO’s composition was analyzed using GC-FID-MS analysis. Fifty different volatile constituents were identified, corresponding to 99.3% of the total constituents in this oil (Table 1). The main group of compounds was oxygenated monoterpenes, mainly alcohols, aldehydes, and esters. Menthone (37.5%), menthol (29.9%), and their isomers such as isomenthone (6.6%) and neomenthol (2.7%) were present in the greatest amounts. A high content of menthyl acetate (9.4%) was also found. The qualitative and quantitative composition complies with the European Pharmacopeia 10.0 [54].

### 2.2. Aphis fabae *Scop.*

**Figure 1:**
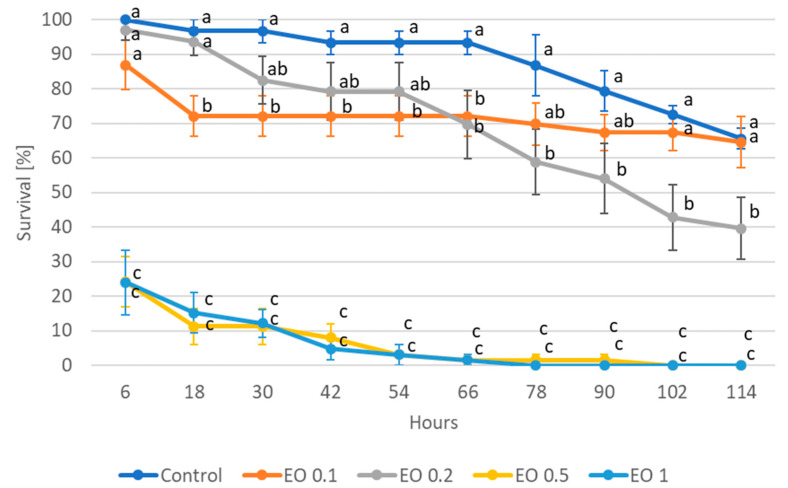
Survival of nymphs of *Aphis fabae* Scop. feeding on mock-orange leaves after the application of peppermint EO in different concentrations. Control—redistilled water; EO 0.1—0.1% concentration of peppermint EO; EO 0.2—0.2% concentration of peppermint EO; EO 0.5—0.5% concentration of peppermint EO; EO 1—1% concentration of peppermint EO. Means ± SE for individual dates of observations marked by different letters are statistically different (*p* ≤ 0.05).

Analysis of variance showed a significant (*p* ≤ 0.05) effect of all applied EO concentrations on the survival of both the nymphs and wingless females of *A. fabae* (Tables S1 and S2). Mortality increased with increasing EO dose, as well as with the duration of the experiment. In the case of nymphs, as early as 6 h after the start of the experiment, EOs at concentrations of 0.5 and 1% caused nearly 80% insect mortality (Figure 1), reaching 100% after 102 h and 78 h, respectively. In turn, doses of 0.1% and 0.2% caused mortality of about 30% after 66 h of the experiment (with 7% mortality in the control), reaching 60% mortality at the end of the experiment (after 114 h) for the 0.2% dose. In contrast, nymph mortality under 0.1% EO after 114 h was not significantly different from the control.

**Figure 2:**
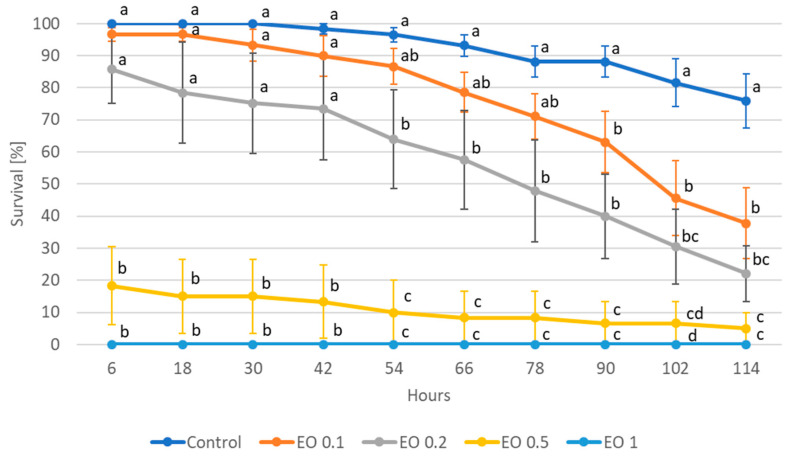
Survival of wingless females of *Aphis fabae* Scop. feeding on mock-orange leaves after the application of peppermint EO in different concentrations. Control—redistilled water; EO 0.1—0.1% concentration of peppermint EO; EO 0.2—0.2% concentration of peppermint EO; EO 0.5—0.5% concentration of peppermint EO; EO 1—1% concentration of peppermint EO. Means ± SE for individual dates of observations marked by different letters are statistically different (*p* ≤ 0.05).

Wingless females proved to be more sensitive to the doses of EO used. After just 6 h, the highest concentration of EO (1%) resulted in 100% mortality, while a dose of 0.5% at that time resulted in 80% mortality (Figure 2). Significant effects of lower doses, i.e., 0.1% and 0.2% were observed after 90 h (37% mortality with 12% mortality in control) and 54 h (36% mortality with 4% mortality in control), respectively. At the end of the experiment (i.e., after 114 h), the 0.2% dose caused 78% mortality of the females of *A. fabae*, while the 0.1% dose caused 62% mortality, respectively. The mortality of control wingless females at this point was 24%. 

The calculated LC_50_ values after 6 h, 30 h, and 78 h for nymphs were 0.5442, 0.3400, and 0.1994%, respectively, while for wingless females at the same times, they were 0.3768, 0.3375, and 0.2063% (Table 2).

### 2.3. Leptinotarsa decemlineata *(Say.)*

The body weight gain of Colorado potato beetle L2 larvae and the mass of eaten food were also significantly (*p* ≤ 0.05) influenced by the application of *M. piperita* EO (Table 3, Tables S3 and S4). A 0.5% concentration of EO and higher caused a decrease in larvae body weight, while a 0.2% concentration had no significant effect on body weight gain for 72 h of the experiment. In contrast, at 96 h of the experiment in this treatment, body weight gain was even significantly greater than in the control. All the EO concentrations used caused a significant reduction in the weight of the food eaten during the 72 h experiment. However, the differences were not statistically significant at a later time (after 96 h). 

Statistical analysis showed no significant effect of the EO concentrations used on body weight gain and mass of eaten food for older larvae, i.e., at the L4 stage (Table 4, Tables S5 and S6). 

**Figure 3:**
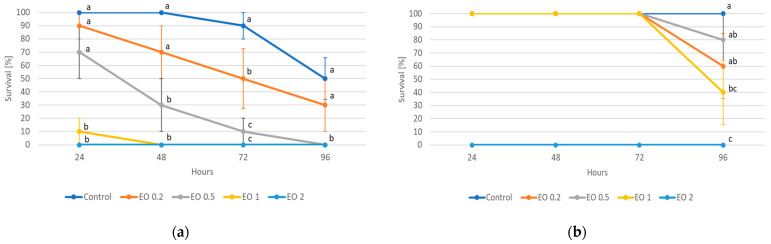
Survival of *Leptinptarsa decemlineata* (Say) 2nd (L2) (**a**) and 4th (L4) instar (**b**) larvae feeding on potato leaves after the application of peppermint EO in different concentrations. Control—redistilled water; EO 0.2—0.2% concentration of peppermint EO; EO 0.5—0.5% concentration of peppermint EO; EO 1—1% concentration of peppermint EO; EO 2—2% concentration of peppermint EO. Means ± SE for individual dates of observations marked by different letters are statistically different (*p* ≤ 0.05). Where letters are not shown, no significant differences were found.

ANOVA showed a significant effect of the EO used on the survival of potato beetle larvae (Figure 3, Tables S7 and S8). A 2% concentration of EO caused 100% mortality of the larvae of both stages (i.e., L2 and L4) as early as in the first hours of the experiment. L2 larvae also died completely when exposed to a 1% concentration of the oil, but only after 48 h (Figure 3a), while such a concentration had no effect on L4 larvae until 96 h into the experiment (Figure 3b). The 0.5% concentration caused a significant increase in the mortality of younger larvae starting from 48 h of the experiment (70% mortality, with 0% mortality in the control), while it had no effect on older larvae. A significant effect of the 0.2% concentration was noted only at 72 h of the experiment, where it caused the death of about half of the individuals tested.

The calculated LC_50_ value for L2 larvae after 1, 2, and 3 days of the experiment was 0.6278, 0.3449, and 0.2020%, respectively (Table 5). For L4 larvae, the LC_50_ value on the fourth day of the experiment was 0.7289%.

### 2.4. Harmonia axyridis *Pallas*

**Figure 4:**
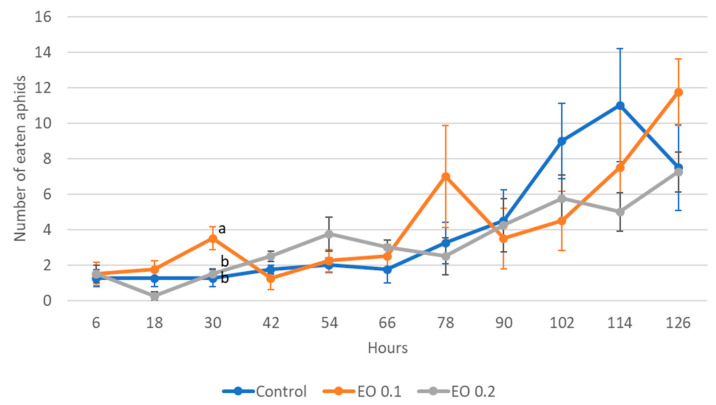
Number of aphids eaten by one 2-day-old larva of *Harmonia axyridis* Pallas in 12 h intervals after the application of the peppermint EO in different concentrations. Control—redistilled water; EO 0.1—0.1% concentration of peppermint EO; EO 0.2—0.2% concentration of peppermint EO. Means ± SE for individual dates of observation marked by different letters are statistically different (*p* ≤ 0.05). Where letters are not shown, no significant differences were found.

**Figure 5:**
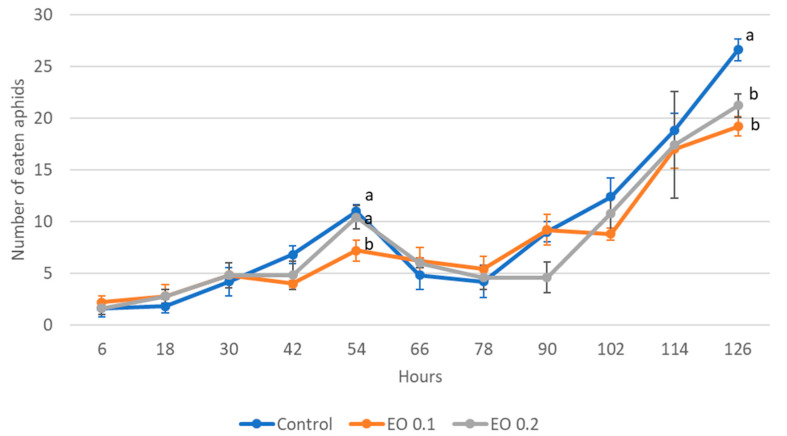
Number of aphids eaten by one 5-day-old larva of *Harmonia axyridis* Pallas in 12 h intervals after the application of the peppermint EO in different concentrations. Control—redistilled water; EO 0.1—0.1% concentration of peppermint EO; EO 0.2—0.2% concentration of peppermint EO. Means ± SE for individual dates of observation marked by different letters are statistically different (*p* ≤ 0.05). Where letters are not shown, no significant differences were found.

**Figure 6:**
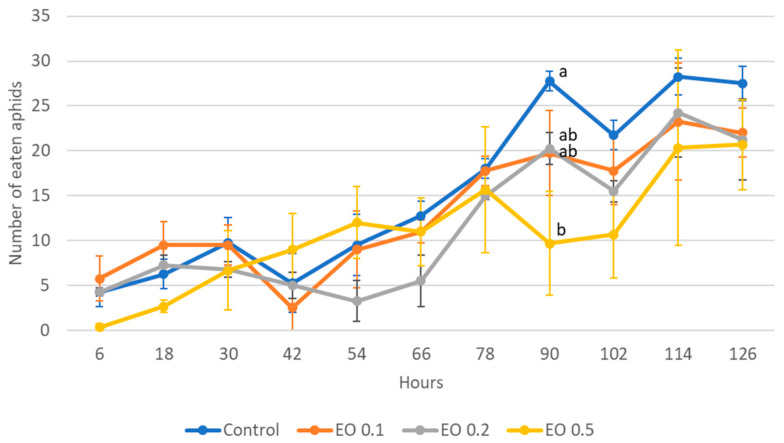
Number of aphids eaten by one 8-day-old larva of *Harmonia axyridis* Pallas in 12 h intervals after the application of the peppermint EO in different concentrations. Control—redistilled water; EO 0.1—0.1% concentration of peppermint EO; EO 0.2—0.2% concentration of peppermint EO; EO 0.5—0.5% concentration of peppermint EO. Means ± SE for individual dates of observation marked by different letters are statistically different (*p* ≤ 0.05). Where letters are not shown, no significant differences were found.

**Figure 7:**
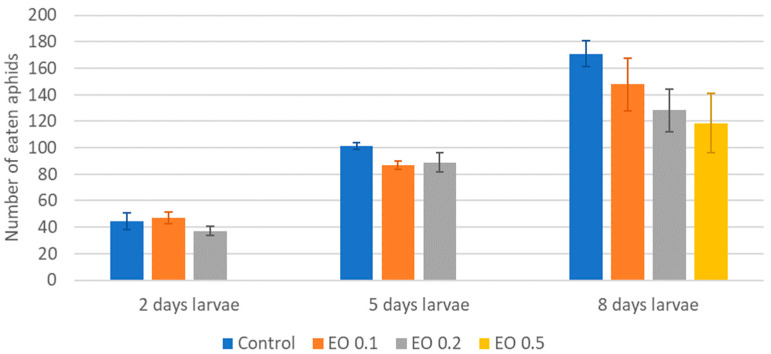
Total number of aphids eaten by one larva of *Harmonia axyridis* Pallas at different ages after the application of the peppermint EO in different concentrations. Control—redistilled water; EO 0.1—0.1% concentration of peppermint EO; EO 0.2—0.2% concentration of peppermint EO; EO 0.5—0.5% concentration of peppermint EO. Means ± SE for individual age group larvae are not statistically different (*p* ≤ 0.05).

The 2-day-old and 5-day-old larvae of the Asian lady beetle died 100% under the influence of oil in concentrations of 0.5 and 1%. Older (8-day-old) larvae, on the other hand, died only under the influence of a 1% concentration. In contrast, at concentrations of 0.2% and 0.1%, all larval stages survived 100%. The oldest larvae, i.e., 8-day-old larvae, also survived 100% at a concentration of 0.5%. A statistical analysis of the voraciousness of Asian lady beetle larvae generally showed no significant effect of EO at concentrations of 0.1, 0.2, and 0.5% (Figure 4, Figure 5 and Figure 6, Tables S9–S11). In the case of the oldest larvae, a significantly lower number of aphids eaten was noticed at 90 h of the experiment in the treatment where 0.5% EO was used (Figure 6). In the case of 5-day-old larvae, on the other hand, fewer aphids being eaten was noticed in 54 h of the experiment in the treatment where 0.1% EO was used, as well as in 126 h of the experiment, and also for the treatment with a 0.2% oil concentration (Figure 5). In turn, the youngest larvae ate even more aphids in 30 h of the experiment in the treatment where 0.1% EO was used than in the control (Figure 4). A comparison of the sum of aphids eaten by ladybug larvae over the course of the experiment showed no significant effect of the tested concentrations of EO with *M. piperita* on this parameter (Figure 7). 

## 3. Discussion

GC-FID-MS analysis of the peppermint EO composition used in the present experiment identified 50 volatile constituents. The main constituents were menthone (37.5%), menthol (29.9%), and their isomers such as isomenthone (6.6%) and neomenthol (2.7%). Menthyl acetate (9.4%) was also quite abundant. A very similar composition of EOs from peppermint was found by Derwich et al. [55], identifying 29 compounds; Sajfrtova et al. [46], identifying 23 components; and Heydari et al. [33], who identified 43 components. Moreover, other studies point to menthol as one of the main components of peppermint EO, with the percentage of this constituent varying depending on the geographic origin of the plant, seasonal variation, genetics, age, harvesting time, and extraction method [26,56,57]. Previous studies indicate that menthol, menthone, and methyl acetate are responsible for the insecticidal activity of EO from *M. piperita* [40]. As shown in some studies, hydrodistillation (which was used in the present experiment), in the case of peppermint, allows the extraction of both the highest yield of volatiles and the highest concentration of volatiles in isolate compared to other extraction methods (supercritical fluid extraction; Soxhlet extraction with ethanol or hexane) [46].

Previous studies have shown the significant insecticidal efficacy of peppermint oil against many storage pests and sanitary insects. In contrast, far fewer studies have examined the use of this EO against crop pests. An important aspect here is the selection of the oil dose, so that, on the one hand, it is characterized by its effectiveness in reducing pests and, on the other hand, it does not exhibit phytotoxicity and is safe for non-target organisms. 

In the present study, the contact effects of four concentrations of EO—0.1, 0.2, 0.5, and 1%—were tested against the nymphs and wingless females of the black bean aphid. A significant difference in efficacy was observed between the 0.2% and 0.5% concentrations. The 0.2% concentration appears to be ineffective enough, while 0.5% EO causes about 80% mortality of nymphs and wingless females as early as 6 h after application. The LC_50_ calculated for nymphs and wingless females at this time was 0.5442% and 0.3768%, respectively. Over time, the LC_50_ value decreases. In the available literature, there are no data about the contact toxicity of peppermint EO against *A. fabae*. The fumigant toxicity (i.e., tested in tightly covered containers) of EOs extracted from five plant species (*Satureja isophylla, Mentha spicata, Mentha piperita, Salvia officinalis*, and *Thymus carmanicus*) against viviparous females of *A. fabae* showed the lowest LC_50_ value (9.29 uL/L of air in a 24 h test) for *M. piperita*. In comparison, the LC_50_ for the other EOs ranged from 10.66 to 41.18 uL/L of air [45]. It is difficult to compare the calculated LC_50_ values for fumigation and contact effects (tested in the present experiment). Although, in the present research, aphid mortality was also tested under laboratory conditions, the Petri dishes used were ventilated. Moreover, immediately after soaking the leaves, they were left for two minutes for the excess solution to drain off in order to avoid the possibility of drowning the test specimens. This, of course, involved the partial evaporation of the EO and the weakening of the fumigant effect. In an open area (crop field), where *A. fabae* feeds, the fumigant effect would also be very limited. The contact effect of peppermint EOs against *A. punicae* tested in a similar manner to this experiment, by immersing eucalyptus leaves in a given oil solution, resulted in an LC_50_ after 24 h = 2.971 μg/mL [32]. This is a slightly lower value than in the present experiment (0.3400% and 0.3375% after 30 h for nymphs and wingless females, respectively), but in the case of this study, the Petri dishes were also wrapped with parafilm, which may have enhanced the fumigant effect. Heydari et al. [33], testing the contact effect of an eco-friendly pesticide based on a peppermint oil nanoemulsion against a cotton aphid, calculated an LC_50_ of 3879.5 μL of active ingredient/L, which is very consistent with the results obtained against *A. fabae* in the present research. The experiment was conducted in a very similar manner to the present one, with ventilation of the dishes. Peppermint EO was listed as one of five that show excellent efficacy in both contact and fumigation tests and can thus be considered an optimal source of active substances against aphids [40]. The authors indicated LD_50_ below 1 μL (μg) mL^−1^ in contact tests, which corresponds to 0.1%, the limit value in their review. The LC_50_ values calculated in this study are slightly higher, but it should be noted that the authors of the studies cited used different solvents (dimethyl sulfoxide (DMSO), acetone, ethanol, n-hexane, and chloroform). Ethanol, in one of these studies, proved to be the most effective solvent [58]. In the present study, the initial dilution (up to 10%) was made with 96% ethanol, while subsequent dilutions were made with redistilled water, which may have affected the obtained results. Interestingly, in the present experiment, wingless females were the stage more sensitive to EO from *M. piperita* than nymphs. Experiments with plant extracts, in which both the adults and nymphs of aphids are taken into consideration, are very rare. Adults of *Aphis gossypii* Glover, *Aphis spiraecola* Patch, and *Myzus persicae* (Sulzer) were more resistant to Tunisian *Melaleuca styphelioides* leaves’ essential oil than nymphs (fumigant and contact effect) [59]. Moreover, water extracts from plants such as *Satureja montana* L. or *Tanacetum vulgare* L. showed higher sensitivity of nymphs than wingless females [60,61]. However, water extract from *M. piperita* showed a similar effect on the mortality of wingless females and nymphs of *A. fabae* [62], and water extract from *Melissa officinalis* L. exhibited (similar to the present experiment) higher efficacy against wingless females than against nymphs [63]. This may indicate a specific effect of plants from the Lamiaceae family and requires further research. The observed effect could be the result of a larger contact area of female bodies with the oil-treated surface, or/and specific physiology and biochemistry, including mechanisms of dealing with xenobiotics, which differ depending on the developmental stage of the insects [64].

The present study showed that L2 potato beetle larvae responded by reducing the weight of food intake and decreasing body weight when exposed to 0.5% and higher concentrations of peppermint EO. In turn, no such effect was observed for older larvae (L4). The survival of L2 larvae also proved to be much more sensitive to different concentrations of the oil than the survival of L4 larvae. Older larvae survived 100% up to 72 h of the experiment with the use of even a 1% concentration of EO, while 100% of L2 larvae at this concentration perished after just 48 h. The higher resistance of L4 potato beetle larvae compared to younger larval stages (as in the present study) was also observed in many earlier studies using other plant extracts [65,66,67,68,69]. The activity of *Piper nigrum* L.-based extract was effective when early instar *L. decemlineata* larvae were targeted. Late instar larvae could be knocked down only with higher concentrations [67]. LD_50_ values for EOs from five different *Satureja* species were higher the older the larvae were (all four instar larvae were tested) [68]. The LD_90_ values for *Heracleum platytaenium* Boiss extract were calculated as 0.345, 0.342, 0.402, and 0.566 mµ L insect^−1^ for first, second, third, and fourth instar larvae of *L. decemlineata*, respectively [69]. Similarly, other researchers have stated that LD_50_ and LD_90_ values increased along with subsequent larval stages of *L. decemlineata* [70]. This could be related to morphological and physiological changes in the beetle larvae during their development.

In the available literature, there is no information on the effect of peppermint EO on the larvae of *L. decemlineata*. Significant acute toxicity of EO from peppermint was demonstrated against adults of *L. decemlineata* with LD_50_ = 38 µg (after 24 h; topical application) [46]. In contrast, against the larvae of *L. decemlineata*, these authors tested EO from savory and thyme, calculating LD_50_ values of 22 µg and 33 µg (after 24 h; topical application), respectively. Different species of peppermint can show the different insecticidal efficacy of the EOs extracted from them. EO from *Mentha longifolia* was found to be effective against adult *L. decemlineata* with an LC_50_ of 3561 ppm [71], which is very close to the value recorded in the present study for L2 larvae after 48 h (0.3449%), while EO from *Mentha spicata* in the same authors’ study was not effective against the pest. 

The results of the present study showed that the mortality of 2- and 5-day-old Asian lady beetle larvae was similar to that of the *A. fabae* aphid, i.e., 100% of these larvae died at a concentration of 0.5%, while all survived at a concentration of 0.2%. Older larvae (8 days old) were more resistant and also survived the 0.5% concentration. There are no data in the available literature on the effects of peppermint EO on the Asian lady beetle. For comparison, the LC_50_ of peppermint oil for third instar larvae of another ladybug species (*Coccinella septempunctata* L. in 24 h toxicity test) was found to be nearly four times higher than that of the aphid *Aphis punicae* [32]. However, the different composition of EOs from peppermint obtained by the cited authors [32] should be emphasized—they identified 17 compounds and the major component of *M. piperita* EO was carvone (61.16%), followed by α-cubebene (10.99%) and D-limonene (4.08%), while the main components of the EOs in the present experiment were menthone (37.5%) and menthol (29.9%). Studies on the effects of EOs from different plants on ladybugs indicate their species-specific responses. In particular, EO extracted from *Thymus capitatus* (L.) Hoffmanns. & Link proved to be effective against the citrus mealybug, *Planococcus citri* Risso, yet the concentrations tested (10 and 20 µL/L-air) showed high toxicity towards coccinellid predator *Cryptolaemus montrouzieri* Mulsant adults [72], which is consistent with the results of this experiment. In contrast, EOs from *Schizogyne sericea* (L.f.) DC. and *Foeniculum vulgare* Miller were not toxic to the third instar larvae and adults of the ladybug *H. axyridis* while being highly effective against the adults of peach-potato aphid *Myzus persicae* Sulzer (LC_50_ of 2.1 mL/L and 0.6 mL/L respectively) [50,51]. However, in the above-mentioned studies, larvae and adult ladybugs, after the application of EOs (by spraying), were placed in clean Petri dishes and then fed with aphids (*M. persicae*) ad libitum. The mortality of the larvae and adults was ascertained after 2 days. In contrast, in this study, *H. axyridis* larvae were fed with aphids that had been treated with EO, so in addition to the contact effect (mock-orange leaf covered with EO solution), the gastric (aphids covered with EO solution) effect may have been important. 

## 4. Materials and Methods

### 4.1. Insect Treatment 

Insects were taken from cultures maintained on purpose for these experiments in the Department of Microbiology and Biomonitoring, University of Agriculture in Krakow. Rearing was carried out using the same plants that were later used in the research. Age and developmental stages were determined by the careful tracking of the development of the individual insects and care was taken to obtain an appropriate number of individuals, uniform in this respect. Bioassay tests were performed on the basis of the modified leaf disc method [73]. 

#### 4.1.1. *Aphis fabae* Scop.

In the case of black bean aphids, mock-orange (*Philadelphus coronarius* L.) leaves were soaked for 3 s in the individual EO solution or in redistilled water (control) and then left at room temperature for 2 min for the excess solution to drain off in order to prevent the possible drowning of the tested insects in EO solutions. Concentrations ranging from 0.1% to 1% were tested against *A. fabae*. The mock-orange leaves were selected to be as similar in size as possible and to come from the same spot on the shoot (to avoid the influence of leaf age on the condition of aphids feeding on them). The test was conducted on Petri dishes (diameter of 9 cm, ventilated to avoid fumigation), and the substrate consisted of moist filter paper. For a given treatment, one leaf was placed per dish, and then pests were introduced—10 individuals per Petri dish. The experiment was conducted separately for wingless females and 6-day-old nymphs. The mortality of the insects was investigated first after 6 h and then every 12 h for five days (the last observation was performed after 114 h). An aphid was considered dead when no leg or antennal movements were observed.

#### 4.1.2. *Leptinotarsa decemlineata* Say.

One larva of the Colorado potato beetle together with one potato leaf (*Solanum tuberosum* L., Bella rosa cultivar, prepared in the same way as the mock-orange leaves) was placed in each Petri dish. The experiment was conducted separately for the L2 (2nd instar) and L4 (4th instar) larvae growth stages. The weight gain of *L. decemlineata*, the mass of eaten food, and mortality were measured 4 times at 24 h intervals. To calculate weight gain, each larva was weighed separately using a precision laboratory balance at the beginning of the experiment and then after subsequent time intervals. The same procedure was applied to the mass of eaten food—each potato leaf was weighed at the beginning of the experiment and then at the same time intervals as the *L. decemlineata* larvae. The mass of eaten food was calculated as the difference between the initial leaf weight and the leaf weight in a given time interval. Loss of leaf weight due to desiccation was taken into account in the calculation. These losses were determined on the basis of weight losses of potato leaves kept in exactly the same conditions as potato leaves in Petri dishes with test insects. Due to the higher resistance of beetle larvae to EO noted in preliminary experiments compared to aphids, concentrations of 0.2% to 2% were tested.

#### 4.1.3. *Harmonia axyridis* Pallas

In the case of *H. axyridis* larvae, mock-orange leaves were used (one leaf per Petri dish, prepared in the same way as for *A. fabae*). Along with the leaf, one lady beetle larva and 15 black bean aphid nymphs of identical size (7 days old) were placed in each dish. The gastric effect of the EO on the voracity of lady beetle larvae as well as mortality were assessed over a period of 126 h. At the start of the experiment and at each subsequent 12 h interval, fresh aphids were added to each Petri dish after determining the number of aphids eaten by noting the number of living and dead aphids. Dead aphids were not included as eaten aphids and they were removed. Aphids were added to restore their number in each Petri dish at the start of each 12 h period. As the larvae grew and their food requirements increased, the number of aphids offered was increased accordingly, so that there was always food in excess. This method was described in an earlier publication [66]. The protocol for each control of the Petri dishes was as follows: after determining the number of eaten aphids, new aphids were added using a fine, moistened brush. Before adding new aphids, they were covered with the specific EO solution or redistilled water by spraying. Care was taken to avoid the drowning or suffocation of the aphids. For ladybug larvae, concentrations of 0.1% to 1% were tested, i.e., the same as for *A. fabae*. *H. axyridis* larvae at different initial ages were selected for the experiments: 2 days old, 5 days old, and 8 days old. 

#### 4.1.4. Experimental Design

The experiments were performed at the University of Agriculture in Krakow in the Department of Microbiology and Biomonitoring under laboratory conditions (at room temperature 24 ± 1 °C in daylight). The experiments were conducted in six replicates and included the following treatments:

C- control—redistilled water;
EO 0.1—0.1% concentration of peppermint EO;
EO 0.2—0.2% concentration of peppermint EO;
EO 0.5—0.5% concentration of peppermint EO;
EO 1—1% concentration of peppermint EO;
EO 2—2% concentration of peppermint EO.

The filter paper in the Petri dishes in all experiments was moistened if necessary to prevent drying the leaves. 

### 4.2. Extraction of EO and Concentrations Preparation

The leaves of *M. piperita*, collected in July 2021 (cultivated at the University of Agriculture in Krakow, southeastern Poland), were used for EO extraction. Leaves were dried at room temperature (26 ± 1 °C) for five days and then were subjected to hydrodistillation using a Clevenger apparatus for 3 h. As a solvent, 96% ethanol was used, which studies indicate yields the best results when using peppermint EO [58]. Measured amounts of the oil were diluted in ethanol to obtain a stock solution—a 10% solution of the EO, which was then diluted with redistilled water to obtain appropriate concentrations of the oil. This approach was used to minimize the negative effect of the solvent on test insects (which was found in preliminary research; the negative effect of ethanol as a solvent on test insects was also noted by other authors [46]). To evaluate the effect of the final solvent concentrations before the actual experiment, preliminary tests were conducted on the effect of the same concentrations of ethanol solutions in redistilled water as those used in the experiment on all test insects. No negative effect of such prepared solvent dilutions on these organisms was demonstrated.

### 4.3. Chemical Composition of EO

The EO was analyzed by gas chromatography coupled with mass spectrometry (GC-FID-MS). Apparatus: Trace GC Ultra gas chromatograph coupled with DSQ II mass spectrometer (Thermo Electron Corporation, Thermo Fisher Scientific Inc., Waltham, MA, USA), non-polar capillary column Rtx-1 ms (60 m × 0.25 mm, 0.25 m film thickness), programmed temperature: 50 (3 min)—300 °C, 4 °C/min, injector (SSL) temperature 280 °C, detector (FID) temperature 300 °C, transfer line temperature 250 °C, carrier gas—helium, flow with constant pressure 200 kPa, split ratio 1:20. The mass spectrometer parameters were ion source temperature 200 °C, ionization energy 70 eV (EI), scan mode: full scan, mass range 33–420. The percentages of constituents were computed from the GC peak area without using a correction factor. The components were identified based on comparing their mass spectra with those reported by Adams (2007) [74] and computer libraries: NIST 2011, and MassFinder 4.1. Additionally, laboratory (RI lab) and literature retention indices (RI lit) collected in the Institute of Natural Products and Cosmetics database of the Lodz University of Technology were compared to confirm identification. The laboratory linear retention indexes were determined regarding a series of n-alkanes C8–C24.

### 4.4. Statistical Analysis

The obtained results were analyzed and checked for normality (Shapiro–Wilk test with Lilliefors correction) and equality of variance (Levene’s test). The significance of differences between the mean values was tested by one-factor variance analysis (STATISTICA 13.0 software), and the mean values were differentiated by Fisher’s LSD test at *p* < 0.05. The StatsDirect software program (Ver. 3.2.7) was used to calculate the probit analyses of LC50 (concentration lethal to half of the test animals) values according to Finney [75].

## 5. Conclusions

Our research indicates promising use for the EO from *M. piperita* against *A. fabae* aphids and young larvae (second instars) of the Colorado potato beetle. *M. piperita* EO showed good insecticidal efficacy against *A. fabae* with LC_50_ = 0.5442% for nymphs and 0.3768% for wingless females after 6 h. Over time, the LC_50_ value decreased. For the L2 larvae of *L. decemlineata*, the LC_50_ values were 0.6278%, 0.3449%, and 0.2020% after 1, 2, and 3 days of the experiment, respectively. There was also a significant reduction in the weight of food eaten and a decrease in the body weight of L2 potato beetle larvae under the influence of EO at concentrations of 0.5% and higher. On the other hand, older larvae (L4) were characterized by significant resistance to the tested oil concentrations with LC_50_ value = 0.7289% after 96 h. Therefore, in the case of the field use of peppermint EO against *L. decemlineata*, it would be recommended to use it against young larvae (LC_90_ after 72 h for L2 = 0.4908%). *M. piperita* EO (contact and gastric effects) at a concentration of 0.5% was found to be toxic to the young larvae (2 and 5 days old) of *H. axyridis*, while EO at a concentration of 1% was toxic to 8-day-old larvae. Thus, for the sake of ladybug safety, it would be advisable to use EO from *M. piperita* against aphids at concentrations lower than 0.5%, although the effect on aphids will not be immediate. Calculated LC_90_ values reached levels below 0.5% both for nymphs and wingless females after 78 h. In the future, it is necessary to test the phytotoxicity of the recommended concentrations for specific target plants.

## Figures and Tables

**Table 1:** The composition of peppermint EO.

No.	Compound	RI _lab_^1^	RI _Lit_^2^	EO ^3^ (%)	EP ^4^ (%)
1	α-Pinene	936	927	0.4	
2	Sabinene	970	962	0.2	
3	β-Pinene	974	966	0.6	
4	Octan-3-ol		979	0.1	
5	Myrcene	987	979	0.1	
6	1,8-Cineol	1024	1016	3.2	3.5–8.0
7	Limonene	1025	1018	0.4	1.0–3.5
8	(*Z*)-β-Ocimene	1029	1025	0.1	
9	(*E*)-β-Ocimene	1034		t ^5^	
10	γ-Terpinene	1055	1048	t	
11	Linalool	1086	1085	0.3	
12	Isopentyl 2-methylbutanoate	1091	1090	t	
13	Isopentyl 2-methylbutanoate	1092	1093	0.1	
14	*trans*-Pinocarveol	1025	1125	t	
15	Menthone	1136	1138	37.5	14.0–32.0
16	Isomenthone	1146	1144	6.6	1.5–10.0
17	δ-Terpineol	1155	1148	t	
18	Neomenthol	1156	1151	2.7	
19	Menthol	1172	1163	29.9	30.0–55.0
20	Isomenthol	1175	1170	0.5	
21	Neoisomenthol	1176	1174	0.2	
22	Pulegone	1215	1214	0.1	max. 3.0
23	Piperitone	1226	1226	0.6	
24	Linalyl acetate	1239	1240	0.1	
25	Isopulegol acetate (isomer I)	1263	1257	t	
26	Neomenthyl acetate	1263	1261	0.1	
27	Thymol	1267	1271	0.1	
28	Menthyl acetate	1280	1280	9.4	2.8–10.0
29	Isomenthyl acetate	1298	1293	0.2	
30	Eugenol	1331	1335	t	
31	α-Copaene	1379	1377	t	
32	α-Elemene	1380	1381	t	
33	β-Bourbonene	1380	1385	0.3	
34	β-Elemene	1389	1388	0.7	
35	(*E*)-β-Caryophyllene	1421	1419	1.9	
36	(*E*)-β-Farnesene	1446	1447	0.1	
37	α-Humulene	1450	1452	0.1	
38	γ-Muurolene	1474	1471	t	
39	Germacrene D	1479	1478	1.9	
40	4-epi-Cubebol	1490	1488	t	
41	Bicyclogermacrene	1494	1492	0.1	
42	γ-Cadinene	1507	1507	t	
43	*cis*-Calacorene	1517	1511	t	
44	δ-Cadinene	1520	1515	0.1	
45	α-Cadinene	1534	1531	t	
46	Spathulenol	1572	1565	0.1	
47	Caryophyllene oxide	1578	1572	0.2	
48	Viridoflorol	1592	1583	0.2	
49	1,10-diepi-Cubenol	1615	1604	t	
50	α-Cadinol	1643	1639	0.1	
	Total identified			99.3	

^1^ RI _lit_—literature retention index; ^2^ RI _lab_—experimental retention index; ^3^ EO—peppermint EO; ^4^ EP—European Pharmacopeia 10.0 requirements [54]; ^5^ t—trace amounts *t* < 0.05.

**Table 2:** The LC_50_, LC_90_, and LC_95_ values of the peppermint EO recorded against nymphs and wingless females of *Aphis fabae* Scop. on selected hours after treatment.

Life Stage	Hours	LC_50_ (%)	95% Confidence Limits		Slope *	(X^2^) **	LC_90_ (%)	LC_95_ (%)
Nymphs			Lower	Upper				
	6	0.5442	0.3832	0.7196	2.0188	68.1927	1.0884	1.2734
	18	0.3942	0.2548	0.5394	2.2052	64.8764	0.8924	1.0618
	30	0.3400	0.2250	0.4567	2.2672	46.1155	0.8246	0.9894
	54	0.2705	0.1381	0.4066	3.9759	141.9055	0.6407	0.5468
	78	0.1994	0.1641	0.2384	5.6428	17.0729 ^1^	0.4603	0.3940
	90	0.1847	0.1481	0.2226	5.5391	17.0622 ^1^	0.3830	0.4505
Wingless females	6	0.3768	0.3067	0.4496	6.1061	58.4319	0.5567	0.6179
	18	0.3523	0.2721	0.4366	6.0243	76.6022	0.5346	0.5966
	30	0.3375	0.2556	0.4248	5.4347	76.3419	0.5396	0.6084
	54	0.2807	0.2061	0.3620	5.1156	67.6146	0.4955	0.5685
	78	0.2063	0.1285	0.2845	4.1734	61.8666	0.4695	0.5590
	90	0.1639	0.0841	0.2368	4.0298	52.3328	0.4365	0.5292

* Slope of the regression line, ** Chi-square value (22 df), *p* < 0.0001, except for ^1^, where *p* > 0.05.

**Table 3:** Body weight gain of *Leptinotarsa decemlineata* (Say) 2nd instar (L2) larvae (mg) after the application of the peppermint EO in different concentrations at 24, 48, 72, and 96 h of the experiment. Control—redistilled water; EO 0.2—0.2% concentration of peppermint EO; EO 0.5—0.5% concentration of peppermint EO; EO 1—1% concentration of peppermint EO; EO 2—2% concentration of peppermint EO; T_0_—body weight of *L. decemlineata* L2 larva at the beginning of the experiment.

Treatment	Exposure Time (h)			
	24	48	72	96
	L2 body weight gain compared to T_0_ (mg)			
Control	15.20 (±3.09) a *	12.30 (±8.63) a	17.40 (±4.31) a	8.60 (±2.62) a
EO 0.2	11.80 (±6.51) a	22.90 (±8.03) a	20.13 (±7.44) a	20.67 (±4.57) b
EO 0.5	−11.60 (±2.48) b	−12.25 (±2.43) b	−12.75 (±0.75) b	−3.00 (±0.00) a
EO 1	−7.30 (±1.71) b	−9.00 (±0.00) ab	-	-
EO 2	−1.60 (±2.90) b	-	-	-
	Mass of leaves eaten by one L2 larva (mg)			
Control	16.97 (±7.56) a	88.25 (±17.26) a	121.60 (±15.71) a	193.72 (±24.85) a
EO 0.2	0.00 b	11.60 (±11.60) b	42.94 (±27.23) b	99.05 (±49.46) a
EO 0.5	0.00 b	0.00 b	8.80 (±8.80) b	18.00 (±0.00) a
EO 1	0.00 b	0.00 b	-	-
EO 2	0.00 b	-	-	-

* Means ± SE for individual dates of observations marked by different letters in columns are statistically different (*p* ≤ 0.05).

**Table 4:** Body weight gain of *Leptinotarsa decemlineata* (Say) 4th instar (L4) larvae (mg) after the application of the peppermint EO in different concentrations at 24, 48, 72, and 96 h of the experiment. Control—redistilled water; EO 0.2—0.2% concentration of peppermint EO; EO 0.5—0.5% concentration of peppermint EO; EO 1—1% concentration of peppermint EO; EO 2—2% concentration of peppermint EO; T_0_—body weight of *L. decemlineata* L4 larvae at the beginning of the experiment.

Treatment	Exposure Time (h)			
	24	48	72	96
	L4 body weight gain compared to T_0_ (mg)			
Control	−20.20 (±5.10) *	−28.20 (±4.71)	−29.00 (±7.06)	−34.60 (±6.59)
EO 0.2	−10.20 (±16.63)	−30.80 (±13.41)	−32.20 (±10.09)	−32.80 (±10.35)
EO 0.5	−19.00 (±13.72)	−30.60 (±7.72)	−33.40 (±8.72)	−37.60 (±7.95)
EO 1	−40.20 (±7.87)	−45.60 (±6.19)	−48.20 (±4.97)	−51.80 (±8.55)
EO 2	−27.80 (±9.16)	-	-	-
	Mass of leaves eaten by one L4 larva (mg)			
Control	0.00	27.62 (±27.62)	71.84 (±38.22)	231.85 (±65.99)
EO 0.2	0.00	112.10 (±73.00)	165.53 (±87.20)	327.63 (±79.23)
EO 0.5	0.00	224.25 (±105.43)	334.76 (±106.54)	463.27 (±103.64)
EO 1	0.00	19.98 (±19.98)	82.18 (±61.11)	365.06 (±87.96)
EO 2	10.61 (±10.61)	-	-	-

* Means ± SE. Letters are not shown because no significant differences were found (*p* ≤ 0.05).

**Table 5:** The LC_50,_ LC_90_, and LC_95_ values of the peppermint EO recorded against 2nd (L2) and 4th (L4) instar larvae of *L. decemlineata* on selected hours after treatment.

Life Stage	Hours	LC_50_ (%)	95% Confidence Limits		Slope *	(X^2^) **	LC_90_ (%)	LC_95_ (%)
L2			Lower	Upper				
	24	0.6278	0.4550	0.8602	2.8184	16.7753	1.0176	1.1502
	48	0.3449	0.1235	0.5161	3.2938	16.4591	0.6973	0.7919
	72	0.2020	−0.5699	0.3407	3.8046	12.7374	0.4908	0.5890
L4	96	0.7289	−0.0543	1.7517	1.1485	17.6508	1.6854	2.0107

* Slope of the regression line, ** Chi-square value (22 df) *p* > 0.05. For other dates (96 h for L2 larvae and 24, 48, and 72 h for L4 larvae) according to the data on the survival of larvae in specific treatments, LC_50,_ LC_90_, and LC_95_ values are not possible to calculate.

## Data Availability

Not applicable.

## Supplementary Materials

The following supporting information can be downloaded at: https://www.mdpi.com/article/10.3390/molecules28124647/s1, Table S1: ANOVA results of the survival of *Aphis fabae* Scop. nymphs; Table S2. ANOVA results of the survival of wingless females of *Aphis fabae* Scop.; Table S3. ANOVA results of the body weight gain of L2 larvae of *Leptinotarsa decemlineata* Say.; Table S4. ANOVA results of the mass of leaves eaten by L2 larvae of *Leptinotarsa decemlineata* Say.; Table S5. ANOVA results of the body weight gain of L4 larvae of *Leptinotarsa decemlineata* Say.; Table S6. ANOVA results of the mass of leaves eaten by L4 larvae of *Leptinotarsa decemlineata* Say.; Table S7. ANOVA results of the survival of L2 larvae of *Leptinotarsa decemlineata* Say.; Table S8. ANOVA results of the survival of L4 larvae of *Leptinotarsa decemlineata* Say.; Table S9. ANOVA results of the number of aphids eaten by one 2-day-old larva of *Harmonia axyridis* Pallas.; Table S10. ANOVA results of the number of aphids eaten by one 5-day-old larva of *Harmonia axyridis* Pallas.; Table S11. ANOVA results of the number of aphids eaten by one 8-day-old larva of *Harmonia axyridis* Pallas.

## Sample Availability

Samples of the compounds are not available from the authors.
