# Supplementary material for: Contact and Gastric Effect of Peppermint Oil on Selected Pests and Aphid Predator Harmonia axyridis Pallas
Source: Molecules. 2023 Jun 8;28(12):4647. doi: 10.3390/molecules28124647 (PMC10302454; doi:10.3390/molecules28124647)
Supplement: Supplementary file 1 [file molecules-28-04647-s001.zip › molecules-2415730-supplementary.pdf]

Table S1. Anova result on the survival of *Aphis fabae* Scop. nymphs.

Hours	Sum of Squares	df	Mean Square	F	p
6h	31409.24	4	7852.310	30.886	0.000000
18h	36113.84	4	9028.460	66.086	0.000000
30h	32330.09	4	8082.523	50.949	0.000000
42h	34892.68	4	8723.169	52.787	0.000000
54h	38002.62	4	9500.654	61.017	0.000000
66h	35687.23	4	8921.807	50.478	0.000000
78h	30306.66	4	7576.664	40.970	0.000000
90h	26229.05	4	6557.262	35.617	0.000000
102h	22782.32	4	5695.581	37.043	0.000000
114h	19776.20	4	4944.049	31.209	0.000000

Table S2. Anova result on the survival of wingless females of *Aphis fabae* Scop.

Hours	Sum of Squares	df	Mean Square	F	p
6h	53647.85	4	13411.96	41.818	0.000000
18h	53350.41	4	13337.60	28.773	0.000000
30h	51066.78	4	12766.69	26.462	0.000000
42h	49233.44	4	12308.36	24.076	0.000000
54h	46735.40	4	11683.85	26.299	0.000000
66h	41660.69	4	10415.17	24.336	0.000000
78h	35418.75	4	8854.69	18.643	0.000000
90h	33359.87	4	8339.97	20.901	0.000000
102h	25778.90	4	6444.73	14.403	0.000003
114h	22438.32	4	5609.58	16.079	0.000001

Table S3. Anova result on body weight gain of L2 larvae of *Leptinotarsa decemlineata* Say.

Hours	Sum of Squares	df	Mean Square	F	p
24h	0.00276	4	0.00069	9.948	0.0001
48h	0.00313	3	0.00104	4.029	0.0369
72h	0.00163	2	0.00081	6.281	0.0229
96h	0.00050	2	0.00025	5.764	0.0401

Table S4. Anova result on the mass of leaves eaten by L2 larvae of *Leptinotarsa decemlineata* Say.

Hours	Sum of Squares	df	Mean Square	F	p
24h	0.00114	4	0.00028	4.731	0.0081
48h	0.02181	3	0.00727	8.404	0.0044
72h	0.02367	2	0.01183	6.767	0.0190
96h	0.03428	2	0.01714	3.805	0.0857

Table S5. Anova result on body weight gain of L4 larvae of *Leptinotarsa decemlineata* Say.

Hours	Sum of Squares	df	Mean Square	F	p
24h	0.00253	4	0.00063	0.992	0.4344
48h	0.00095	3	0.00032	0.844	0.4895
72h	0.00109	3	0.00036	1.155	0.3574
96h	0.00112	3	0.00037	1.039	0.4021

Table S6. Anova result on the mass of leaves eaten by L4 larvae of *Leptinotarsa decemlineata* Say.

Hours	Sum of Squares	df	Mean Square	F	p
24h	0.00045	4	0.00011	1.000	0.4307
48h	0.13581	3	0.04527	2.057	0.1464
72h	0.22175	3	0.07392	2.449	0.1012
96h	0.13740	3	0.04580	1.259	0.3219

Table S7. Anova result on survival of L2 larvae of *Leptinotarsa decemlineata* Say.

Hours	Sum of Squares	df	Mean Square	F	p
24h	42600	4	10650	17.750	0.000002
48h	39000	4	9750	12.188	0.000035
72h	31000	4	7750	11.071	0.000067
96h	10600	4	2650	4.077	0.014126

Table S8. Anova result on survival of L4 larvae of *Leptinotarsa decemlineata* Say.

Hours	Sum of Squares	df	Mean Square	F	p
96h	29600	4	7400	4.625	0.0083

Table S9. Anova result on the number of aphids eaten by one 2-days larvae of *Harmonia axyridis* Pallas.

Hours	Sum of Squares	df	Mean Square	F	p
6h	0.167	2	0.083	0.070	0.933
18h	4.667	2	2.333	3.360	0.081
30h	12.167	2	6.083	6.257	0.020
42h	3.167	2	1.583	2.192	0.168
54h	7.167	2	3.583	1.843	0.213
66h	3.167	2	1.583	1.036	0.394
78h	46.500	2	23.250	1.638	0.247
90h	2.167	2	1.083	0.099	0.907
102h	43.167	2	21.583	1.803	0.220
114h	72.667	2	36.333	1.124	0.367
126h	51.167	2	25.583	1.820	0.217
Total	205.167	2	102.583	1.013	0.401
Mean	1.696	2	0.848	1.013	0.401

Table S10. Anova result on the number of aphids eaten by one 5-days larvae of *Harmonia axyridis* Pallas.

Hours	Sum of Squares	df	Mean Square	F	p
6h	1.200	2	0.600	0.265	0.772
18h	3.333	2	1.667	0.472	0.635
30h	1.200	2	0.600	0.075	0.928
42h	20.800	2	10.400	2.328	0.140
54h	41.733	2	20.867	4.637	0.032
66h	5.733	2	2.867	0.455	0.645
78h	3.733	2	1.867	0.217	0.808
90h	67.600	2	33.800	3.756	0.054
102h	32.533	2	16.267	1.406	0.283
114h	8.933	2	4.467	0.082	0.922
126h	146.533	2	73.267	13.162	0.001
Total	601.733	2	300.867	2.540	0.120
Mean	4.973	2	2.487	2.540	0.120

Table S11. Anova result on the number of aphids eaten by one 8-days larvae of *Harmonia axyridis* Pallas.

Hours	Sum of Squares	df	Mean Square	F	p
6h	52.817	3	17.606	1.746	0.215
18h	82.167	3	27.389	2.297	0.134
30h	31.767	3	10.589	0.420	0.742
42h	72.650	3	24.217	0.813	0.513
54h	150.650	3	50.217	1.067	0.403
66h	118.250	3	39.417	1.824	0.201
78h	25.917	3	8.639	0.278	0.840
90h	561.083	3	187.028	3.904	0.040
102h	221.233	3	73.744	2.297	0.134
114h	114.017	3	38.006	0.267	0.847
126h	113.583	3	37.861	0.782	0.528
Total	5872.767	3	1957.589	1.805	0.204
Mean	48.535	3	16.178	1.805	0.204
